# A structural network fingerprint of mild traumatic brain injury: a multi-study synthesis of T1-weighted MRI abnormalities

**DOI:** 10.3389/fnhum.2026.1800956

**Published:** 2026-03-13

**Authors:** Ioannis Mavroudis, Foivos Petridis, Alin-Stelian Ciobîcă, Roxana O. Cojocariu, Dimitrios Kazis, Ahmed Adel Mansour Kamar, Cătălina Ionescu, Diana Gheban, Catalin Morosan, Bogdan Gurzu, Otilia Novac, Bogdan Novac

**Affiliations:** 1 Leeds Teaching Hospitals, NHS Trust, Leeds, United Kingdom; 2 Romanian Academy of Scientists (Academia Oamenilor de Știință din România), Bucharest, Romania; 3 Third Department of Neurology, Aristotle University of Thessaloniki, Thessaloniki, Greece; 4 Department of Biology, Faculty of Biology, “Alexandru Ioan Cuza” University of Iași, Iași, Romania; 5 “Ioan Hăulică” Institute, Apollonia” University of Iași, Iași, Romania; 6 Biomedical Research Group, “Olga Necrasov” Center, Romanian Academy, Iași, Romania; 7 CENEMED Platform for Interdisciplinary Research, University of Medicine and Pharmacy, “Grigore T. Popa”, Iași, Romania; 8 Department of Biological and Morphological Sciences, Faculty of Medicine and Biological Science, Stefan cel Mare University of Suceava, Suceava, Romania; 9 Medical Department, GUPCO, Cairo Office, Cairo, Egypt; 10 Department of Orthopedics and Traumatology, Clinical Recovery Hospital (Recuperare), Iași, Romania; 11 Doctoral School of Biology, Faculty of Biology, “Alexandru Ioan Cuza” University of Iași, Iași, Romania; 12 Faculty of Medicine, Grigore T. Popa University of Medicine and Pharmacy, Iași, Romania

**Keywords:** default mode network, graph theory, mild traumatic brain injury (mTBI), network topology, neuroimaging synthesis, structural network fingerprint, T1-weighted MRI, thalamo-callosal relay

## Abstract

**Background:**

Mild traumatic brain injury (mTBI) often results in persistent cognitive and somatic deficits despite unremarkable routine neuroimaging. Evidence suggests mTBI affects large-scale neural systems rather than isolated regions, yet structural findings remain heterogeneous across studies.

**Objective:**

This study synthesized T1-weighted MRI data into a unified structural network fingerprint (SNF) of mTBI.

**Methods:**

We analyzed ten peer-reviewed studies identifying regional abnormalities in adult mTBI via voxel-based, volumetry, grey/white-matter probability mapping or tensor-based morphometry. Thirty-five significant regions of interest (ROIs) were extracted and mapped to a standardized anatomical atlas. ROIs were categorized into canonical networks, and we applied co-alteration graph modeling, principal component analysis (PCA), and hierarchical clustering to evaluate network-level convergence.

**Results:**

The SNF identified a core triad of vulnerability: the default mode network (DMN), the limbic/memory system, and thalamic–callosal relay structures. Graph modeling revealed robust clustering among DMN–limbic–thalamic regions. Furthermore, PCA and hierarchical clustering demonstrated that structural alterations strictly align with intrinsic network boundaries, rather than appearing as stochastic damage.

**Conclusion:**

mTBI exhibits a reproducible structural signature characterized by DMN and thalamo-limbic involvement. This SNF framework establishes a basis for clinically interpretable biomarkers and computable decision-support tools in concussion care.

## Introduction

Mild traumatic brain injury (mTBI) and concussion are highly prevalent worldwide, with disproportionately elevated incidence in military personnel, athletes, and young adults (Cassidy et al., 2004; Lishman, 1988; McCrory et al., 2017). Although many individuals experience clinical recovery, a substantial subset continues to report persistent post-concussive symptoms. These commonly encompass cognitive slowing, memory difficulties, chronic headache, and affective dysregulation (Bigler, 2013; Shenton et al., 2012). A central challenge in the clinical management of mTBI lies in its limited visibility on routine diagnostic imaging. Standard computed tomography (CT) and conventional magnetic resonance imaging (MRI) sequences frequently fail to reveal overt structural abnormalities, creating a gap between patients’ subjective symptom burden and objective radiological findings (Yuh et al., 2014; Wintermark et al., 2015). This diagnostic gap not only complicates long-term prognosis but also creates significant hurdles in legal and occupational medicine. Consequently, there is an urgent clinical imperative to identify more sophisticated, quantitative MRI signatures that can capture the subtle structural rearrangements occurring at the network level.

Over approximately the past two decades, conceptual models of traumatic brain injury have evolved from a focus on discrete focal lesions to a recognition of mTBI as a disorder affecting distributed brain networks (Sharp et al., 2014; Hillary et al., 2014; van der Horn et al., 2016). Biomechanical modeling and neuropathological findings suggest that rotational acceleration, axonal shear, metabolic crisis, and neuroinflammatory responses preferentially involve midline and paralimbic regions, long-range association fibers, and highly interconnected hub regions with substantial metabolic demands (Giza and Hovda, 2014; Werner and Engelhard, 2007; Barkhoudarian et al., 2011). Prominent large-scale networks—such as the default mode network (DMN), salience network, and frontoparietal control network (FPN)—are essential for self-referential cognition, memory, emotional regulation, and executive function (Raichle, 2015; Buckner et al., 2008; Seeley et al., 2007), capacities that are commonly impaired in post-concussive syndrome (PCS) (Mayer et al., 2017; Zhou et al., 2013; Collins et al., 2016). In line with these observations, functional MRI and diffusion-based approaches have repeatedly demonstrated network-level alterations in connectivity and microstructure following mTBI (Sharp et al., 2014; Hillary et al., 2014; Mayer et al., 2012; King et al., 2019).

In contrast, findings from structural MRI studies in mTBI have been markedly heterogeneous. Voxel-based morphometry (VBM) investigations have reported grey matter (GM) reductions across a range of regions, including the frontal lobes, cingulate cortex, temporal areas, precuneus, subcortical nuclei, and cerebellum (Zhou et al., 2013; Mavroudis et al., 2022). Volumetric analyses have similarly described atrophy within the thalamus, hippocampus, and other limbic structures (Niu et al., 2020; Kriukova et al., 2024). Conversely, other studies have identified regional increases in GM volume, which have been interpreted as reflecting neuroinflammatory processes, compensatory plasticity, or methodological factors (Niu et al., 2020; Burrowes et al., 2020). This variability is partly attributable to differences in cohort composition, post-injury timepoints, analytic strategies, and statistical thresholds. However, it also reflects a broader limitation of the field, in which structural abnormalities are most often reported at the level of isolated regions rather than within an integrated network context.

Accordingly, there is a clear need for an integrative framework capable of aggregating T1-weighted structural MRI findings across independent mTBI studies, harmonising results within a shared anatomical and network-based space, and quantifying patterns of convergence, co-alteration, and network-level vulnerability. Such an approach is not intended to supplant established meta-analytic methods, such as activation likelihood estimation (ALE) (Mavroudis et al., 2022), but rather to complement them by retaining study-level information, directionality of effects, and underlying network organization. Importantly, a framework grounded in named anatomical regions and canonical brain networks aligns naturally with clinical workflows and with automated neuroimaging analysis pipelines.

Importantly, the present Structural Network Fingerprint (SNF) framework differs conceptually and methodologically from coordinate-based voxel-wise meta-analyses such as activation likelihood estimation (ALE). ALE and related techniques estimate the spatial convergence of reported peak coordinates across studies, generating probabilistic maps of anatomical overlap. While highly valuable, such approaches do not preserve study-level abnormality profiles, directional information (increase versus decrease), or explicit large-scale network organization. In contrast, the SNF framework operates in region-by-study matrix space, retaining directionality of structural changes, mapping regions onto canonical functional networks, and enabling quantification of network-level convergence and co-alteration structure. Rather than replacing conventional meta-analytic methods, SNF provides a complementary systems-level representation of structural vulnerability, emphasizing reproducible network topology over spatial peak density.

In this study, we propose a structural network fingerprint (SNF) framework for mTBI derived exclusively from T1-weighted structural MRI. We identified ten independent T1-based structural studies of adult mTBI, comprising five primary voxel-based morphometry or volumetric investigations included in a recent meta-analysis (Mavroudis et al., 2022), together with five additional cross-sectional and longitudinal studies employing T1-based morphometric approaches (Zhou et al., 2013; Dean and Sterr, 2015; Muller et al., 2021; Kim et al., 2021). From each study, we extracted all brain regions showing statistically significant structural differences in grey or white matter between mTBI and comparison groups, or between clinically defined subgroups. These regions were then mapped onto a standard anatomical atlas and assigned to canonical large-scale brain networks, with the direction of effect (increase versus decrease) explicitly encoded. The resulting data were assembled into a region-by-study matrix, which formed the basis for defining the SNF and for subsequent analyses using network metrics, graph-based models, principal component analysis (PCA), and hierarchical clustering to characterize a structural network signature of mTBI.

By restricting the analysis to T1-weighted structural MRI—including VBM, volumetric measures, grey and white matter probability maps, and tensor-based morphometry—we avoid conflating fundamentally different imaging modalities and remain closely aligned with methods that are widely accessible in clinical practice. The overarching aims of this work were to determine whether a reproducible network-level structural pattern emerges across heterogeneous mTBI cohorts, and to introduce a mathematically explicit framework that can be implemented in software to derive individual-level network-based injury indices.

## Methods

### Study identification and inclusion criteria

The present study focused exclusively on T1-weighted structural magnetic resonance imaging (MRI). Studies were considered eligible if they met the following criteria: (i) inclusion of adult participants with mild traumatic brain injury (mTBI) or sport-related concussion defined according to established diagnostic standards; (ii) acquisition of T1-weighted MRI data with analysis of regional brain structure using voxel-based morphometry (VBM), volumetric approaches, cortical or subcortical morphometry, grey or white matter probability segmentation, or tensor-based morphometry (TBM); and (iii) reporting of anatomically specific regions or clusters demonstrating statistically significant structural differences relative to healthy control groups or between clinically defined subgroups. Studies relying exclusively on diffusion tensor imaging, functional MRI, magnetic resonance spectroscopy, or non–T1-weighted structural contrasts were excluded.

Based on a recent systematic voxel-based meta-analysis of grey matter alterations in mTBI (Gale et al., 2005), we identified five primary T1-weighted structural MRI studies (Gale et al., 2005; Niu et al., 2020; Wilke et al., 2018; Burrowes et al., 2020; Wu et al., 2014). Importantly, activation likelihood estimation (ALE) clusters reported in the meta-analysis were not treated as independent observations; instead, the original T1-based regional findings were extracted directly from each primary publication. This dataset was supplemented with five additional T1-weighted structural MRI studies that were not included in the meta-analysis but satisfied the inclusion criteria: Zhou et al. (2012), Dean and Sterr (2015; grey matter proportion), Muller et al. (2021), Zhou et al. (2012), and Kim et al. (2021). Together, these sources yielded a total of ten independent T1-weighted structural MRI studies included in the present analysis.

### Extraction and coding of regions of interest

For each included study, the full text, tables, and relevant figures were systematically reviewed to identify all brain regions exhibiting statistically significant structural abnormalities. In voxel-based morphometry (VBM) studies, anatomical labels assigned by the original authors were used to define significant clusters. In volumetric and tensor-based morphometry (TBM) studies, regions of interest were defined according to the reported anatomical structures (e.g., thalamus, hippocampus, medial orbitofrontal cortex). When multiple significant clusters were reported within the same anatomical region at the atlas level, these were consolidated into a single region of interest (ROI) for the purposes of the present analysis.

Each ROI was assigned a standardized anatomical label and included in the set of all unique regions identified across studies. For each region, we recorded hemisphere (left, right, or bilateral), tissue type (grey matter, white matter, or mixed in the case of certain brainstem regions), and network membership. ROIs were assigned to one or more canonical large-scale brain networks, reflecting established functional and anatomical organization. The network categories included the default mode network (DMN), salience network, frontoparietal control network (FPN), limbic/memory network, sensorimotor/dorsal attention network, interhemispheric (callosal) system, and brainstem/cerebellar system.

Network assignment was guided primarily by the canonical large-scale cortical network framework described by Yeo et al. (2011), supplemented by structural connectivity literature for subcortical and white-matter regions. Specifically, the precuneus was assigned primarily to the DMN; the anterior insula to the salience network; the superior and middle frontal gyri to the FPN; the hippocampus and amygdala to the limbic/memory network; the thalamus to a limbic/relay network; the corpus callosum to the interhemispheric system; and the cerebellar cortex to the brainstem/cerebellar system (Raichle, 2015; Buckner et al., 2008; Seeley et al., 2007; Cavanna and Trimble, 2006; Leech and Sharp, 2014; Catani and Thiebaut de Schotten, 2008). Regions known to span multiple functional systems—such as the rostral anterior cingulate cortex—were assigned to more than one network.

Network assignments were based on established large-scale functional network frameworks derived from convergent functional MRI and structural connectivity literature. While anatomical regions may participate in multiple overlapping systems, assignment was guided by dominant functional affiliation as described in prior canonical network models. In cases of known multi-network participation (e.g., rostral anterior cingulate cortex), regions were permitted dual assignment to preserve biological plausibility.

We acknowledge that network parcellation schemes vary across the literature; however, the primary objective was not to test a specific parcellation model but to evaluate whether structural abnormalities converge within broadly accepted large-scale systems. Sensitivity analyses exploring alternative grouping strategies are reported below.

### Directional coding of structural abnormalities

For each brain region r and each study s∈S (where S denotes the set of ten included studies), we recorded the direction of the reported structural abnormality relative to the appropriate control or reference group. Reductions identified through volumetric analyses or voxel-based morphometry (VBM)—such as decreased grey matter volume, reduced grey matter probability, or tensor-based morphometry–indicated contraction—were coded as negative changes. Conversely, increases in structural measures (e.g., regional grey matter enlargement) were coded as positive changes. We did not attempt to harmonise absolute effect sizes across studies, as variability in preprocessing pipelines, normalization strategies, and statistical modeling precludes direct comparability across datasets.

Formally, we constructed a structural fingerprint matrix X∈R^∣R∣ × ∣S∣^ where rows correspond to anatomical regions and columns correspond to studies. Matrix entries were defined as:

X(r,s) = − 1, if region r shows a significant decrease in study s.

X(r,s) = + 1, if region r shows a significant increase in study s.

X(r,s) = 0, if region r is not reported as abnormal in study s.

Under this representation, each row of X encodes the abnormality profile of a given region across studies, while each column captures a study-specific pattern of T1-based structural abnormalities.

The decision to encode structural abnormalities using a ternary scheme (−1, 0, +1) reflects both methodological heterogeneity across the included studies and the conceptual aims of the present framework. Differences in preprocessing pipelines, spatial normalization procedures, smoothing kernels, statistical thresholds, and volumetric modeling strategies preclude direct comparison or pooling of reported effect sizes. Accordingly, we adopted a conservative directional encoding that preserves whether a region was reported as significantly decreased or increased, without imposing artificial quantitative comparability across datasets.

This representation is not intended to approximate magnitude of effect, but rather to capture reproducible patterns of regional involvement and directional consistency across independent cohorts. By privileging convergence structure over effect size aggregation, the SNF framework emphasizes topological organization of abnormalities rather than parametric meta-analytic estimation.

### Network-level convergence metrics

To quantify network-level involvement, the region-by-study matrix was expanded according to predefined functional network assignments. For each canonical network k∈K, we defined the corresponding set of regions as Rk​ = {r∈R:k∈N(r)}, where N(r) denotes the set of networks to which region r is assigned.

### Total abnormal load

The total abnormal load for network k was defined as L_k_ = (r∈R_k_) ∑(s∈S)∑∣X(r,s)∣.

This metric reflects how frequently regions belonging to network k are reported as structurally abnormal across all studies, irrespective of the direction of change.

### Number of distinct abnormal regions

The number of distinct abnormal regions within network k was defined as N_k_ = ∣{r∈R_k_:∃s∈S such that X(r,s) ≠ 0}∣.

This measure captures the spatial extent of network involvement by counting how many unique regions within a network show abnormalities in at least one study.

### Normalised abnormality-per-region index

To account for differences in network size, we computed a normalised abnormality-per-region index A_k_ = L_k_/N_k_. This index reflects the consistency with which regions within a given network are reported as abnormal across studies.

### Mean direction of abnormality

To characterize whether network-level abnormalities predominantly reflect reductions or increases in structure, we defined the mean direction of abnormality as D_k_ = 1/L_k_ (r∈R_k_)∑(s∈S)∑X(r,s).

Values of D_k_ approaching −1 indicate predominantly volume loss, values approaching +1 indicate predominantly volume increases, and values near zero reflect mixed or heterogeneous directions of change.

Together, these complementary metrics quantify the frequency, spatial extent, consistency, and directionality of structural abnormalities at the network level.

### Co-alteration graph

To identify regions that tend to be reported as abnormal together across studies, we constructed a co-alteration matrix C∈N^∣R∣ × ∣R∣.^

Each matrix element was defined as C(r1,r2) = (s∈S)∑I[X(r1,s) ≠ 0∧X(r2,s) ≠ 0], where I[·] denotes the indicator function, equal to 1 when the condition is satisfied and 0 otherwise. This value represents the number of studies in which both regions r1 and r2 were reported as structurally abnormal.

Based on this matrix, we defined an undirected co-alteration graph G = (V, E) where nodes V correspond to regions (V = RV) and an edge between regions r1 and r2 exists if C (r1, r2) ≥ *τ*. We set the threshold *τ* = 2 corresponding to co-occurrence in at least two independent studies, in order to emphasize robust and reproducible associations. The choice of *τ* = 2 (co-occurrence in at least two independent studies) was selected as a minimal reproducibility threshold. A threshold of *τ* = 1 would reflect any single-study co-reporting and would therefore be highly sensitive to idiosyncratic findings, while more stringent thresholds (e.g., *τ* ≥ 3) substantially increase sparsity and risk excluding potentially meaningful but less frequently reported associations given the modest number of included studies. Thus, *τ* = 2 represents a balance between sensitivity and robustness, ensuring that retained edges reflect replication across independent cohorts while preserving sufficient graph density to evaluate modular organization.

Edge weights were given by C(r1, r2) and nodes were colour-coded according to their dominant network affiliation. This graph-based representation highlights modular patterns of structural co-alteration as well as cross-network “bridge” regions within the structural network fingerprint.

### Principal component analysis and hierarchical clustering

To examine global patterns within the structural network fingerprint, principal component analysis (PCA) was applied to the region-by-study matrix X following row-wise centering. Specifically, the mean value of each row was subtracted to obtain a centred matrix X, thereby emphasizing relative abnormality profiles across studies rather than absolute reporting frequency.

The covariance matrix was computed as:


Σ=1/(∣S∣−1)XX

Eigen decomposition was then performed according to Σ v_i_ = *λ*_i_ v_i_,

Where v_i_ denotes the eigenvectors defining the principal components and *λ*_i_ corresponding eigenvalues representing the proportion of explained variance. Brain regions were subsequently projected onto the first two principal components to obtain a low-dimensional embedding of structural abnormality patterns, with points coloured according to canonical network membership.

For hierarchical clustering, each row of X was treated as a feature vector in R^∣S∣^. Pairwise Euclidean distances between regions were computed, and agglomerative hierarchical clustering using Ward’s linkage criterion was applied to generate a dendrogram of regions. This approach allowed us to assess whether data-driven groupings of regions corresponded to established large-scale network assignments.

All analyses were implemented in Python using standard scientific computing libraries within a Google Colab environment, supporting reproducibility and straightforward extension to future datasets, including potential patient-level implementations.

## Results

### Overall pattern of T1 structural abnormalities

Across the ten included T1-weighted mTBI studies, approximately 35–40 distinct atlas-defined regions of interest (ROIs) were identified as exhibiting statistically significant structural abnormalities. Early voxel-based morphometry work by Gale et al. (2005) reported grey-matter (GM) reductions involving frontal and temporal cortices, the cingulate gyrus, subcortical GM, and the cerebellum in individuals with mild or mixed-severity traumatic brain injury. In contrast, Niu et al. (2020) observed increased cortical volume in the right dorsal anterior cingulate and right dorsal posterior cingulate cortices in patients with mTBI and chronic post-traumatic headache. Zhuo et al. (Gale et al., 2005) documented volume loss affecting the precuneus and medial orbitofrontal cortex, together with accelerated hippocampal atrophy in individuals sustaining recurrent mTBI. Burrowes et al. (2020) demonstrated GM volume reductions in anterior parietal and temporal opercular regions, the superior and middle frontal gyri, and the left thalamus in mTBI patients both with and without post-traumatic headache.

The remaining five T1-weighted studies corroborated and expanded these observations. Zhuo et al. (2021) reported longitudinal volume reductions involving rostral anterior cingulate white matter, the isthmus cingulate, precuneus, cuneus, and distributed frontal regions over the first year following mTBI. Dean and Sterr (2015) identified reduced GM proportion in the left frontal gyrus, right middle frontal gyrus, left medial temporal lobe, and left parietal cortex, with greater reductions associated with increased post-concussive symptom severity. Using GM and WM probability maps, Muller et al. (2021) demonstrated one-year reductions in the right superior and middle frontal gyri, precentral gyrus, anterior insula, Rolandic operculum, and superior temporal pole, alongside white matter probability decreases in the superior longitudinal fasciculus, frontal aslant tract, frontopontine tract, arcuate fasciculus, external capsule, and corticospinal tract. Zhuo et al. (2021) documented progressive volume loss in the thalamus and hippocampus during the months following mTBI, while Kim et al. (2021) reported tensor-based morphometry–derived volume reductions in the pontine reticular formation in individuals with chronic mTBI.

**Figure 1:**
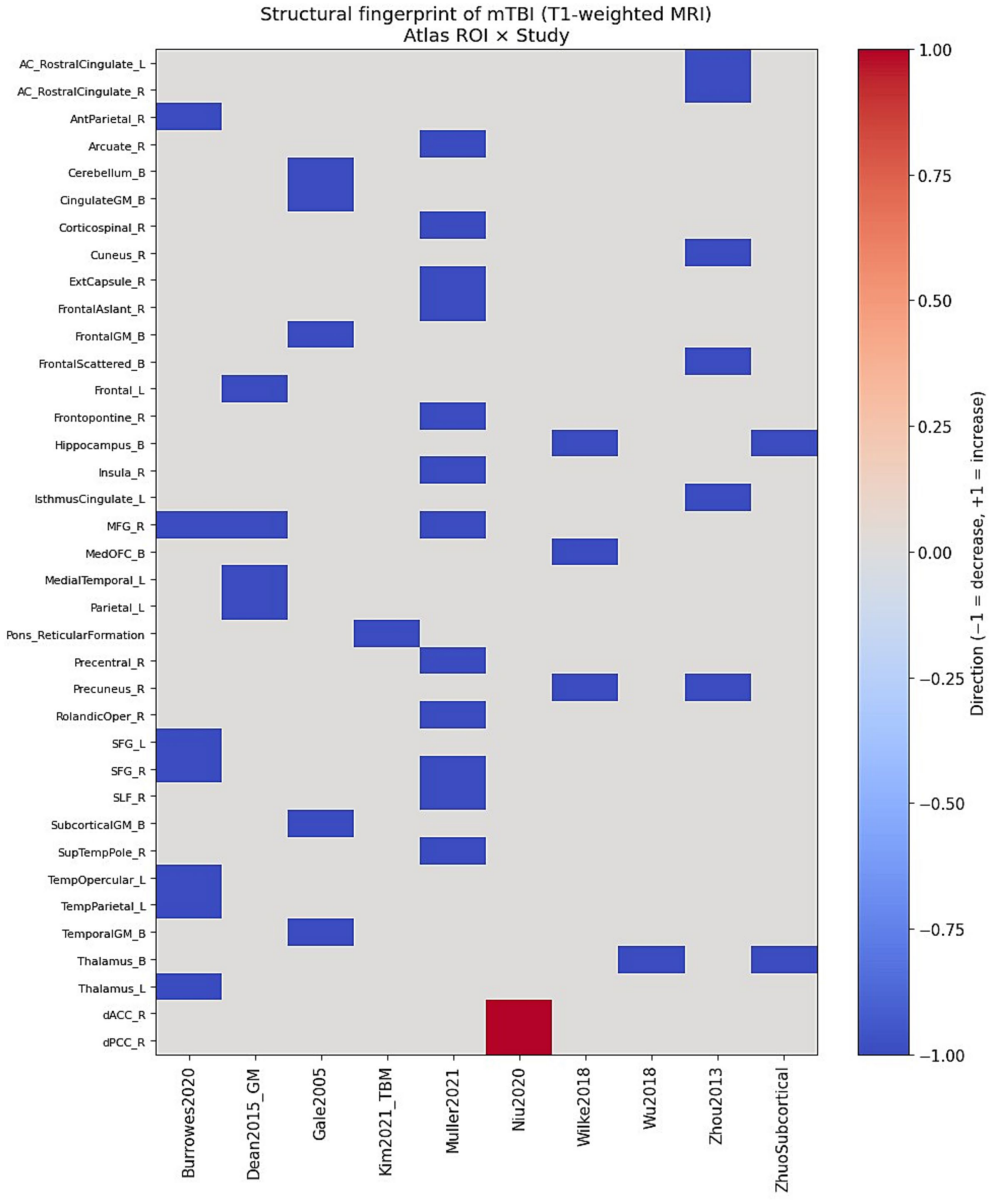
Structural fingerprint matrix of mTBI derived from T1-weighted MRI. This matrix visualization corresponds directly to the structural fingerprint matrix X defined in Methods, with rows representing atlas-based regions, columns representing studies, and color encoding the direction of reported structural abnormalities. Heatmap showing the presence and direction of regional structural abnormalities extracted from ten independent T1-based structural MRI studies of mTBI. Rows represent anatomical regions of interest (ROIs) and columns represent individual studies. Colors encode direction of abnormality: red (−1) indicates reduced grey or white matter volume/probability; blue (+1) indicates increased regional volume; white (0) indicates no reported abnormality. Despite methodological heterogeneity across studies (VBM, volumetry, GM/WM probability maps, TBM), a reproducible pattern of abnormalities emerges, particularly within medial prefrontal, cingulate, precuneus, temporal, thalamic, and hippocampal regions. This matrix forms the basis of the structural network fingerprint (SNF).

Taken together, these findings reveal a distributed pattern of structural vulnerability involving midline and paralimbic regions, frontoparietal association cortex, subcortical relay nuclei, and brainstem structures (Figure 1).

### Network-level convergence

Application of the network-level metrics revealed that three systems carried the highest *cross-study abnormality load across the included cohorts*: the default mode network (DMN), the limbic/memory network, and a combined frontoparietal–salience network complex. Regions associated with the DMN—including the precuneus, posterior and mid cingulate cortex, medial orbitofrontal cortex, and medial temporal structures—were repeatedly identified as abnormal across multiple studies and predominantly exhibited volume reductions, reflected in consistently negative values of the mean direction metric (Dk < 0).

**Figure 2:**
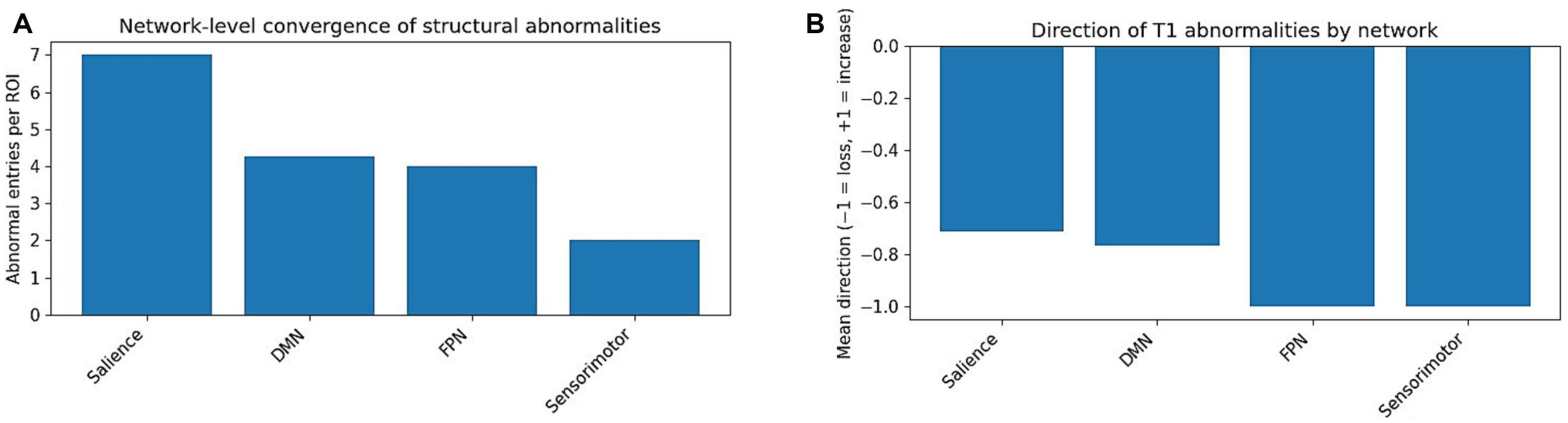
**(A)** Network-level convergence (abnormal entries per ROI). Bar plot showing the ratio of abnormal findings per ROI within each large-scale network. For each canonical network, the number of abnormal entries across all studies was divided by the number of distinct ROIs assigned to that network. Higher values indicate greater vulnerability and more consistent involvement across studies. The default mode network (DMN), limbic/memory network, and frontoparietal/salience networks show the strongest convergence, highlighting them as core structural targets in mTBI. Sensorimotor, interhemispheric, and brainstem/cerebellar networks contribute secondary but meaningful involvement. **(B)** Direction of abnormalities by network. Bar plot illustrating the mean direction of abnormalities within each network. Values range from −1 (consistent volume loss) to +1 (consistent volume increase). The DMN, limbic/memory, and frontoparietal networks show predominantly negative directionality, reflecting grey-matter and white-matter loss across multiple independent studies. Small positive values in some networks reflect isolated reports of increased regional volume, primarily in dorsal anterior and posterior cingulate cortex. Together, this pattern reinforces a signature of structural degeneration in network hubs crucial for attention, memory, and self-regulation.

At the network level, convergence metrics revealed that DMN, limbic/memory, and frontoparietal–salience networks carried the highest abnormality burden (Figure 2A), with predominantly negative mean direction values indicating volume loss (Figure 2B).

Limbic and subcortical regions, most notably the hippocampus and thalamus, showed convergent volume loss across several independent cohorts, particularly in longitudinal and chronic mTBI samples (Burrowes et al., 2020; Dean and Sterr, 2015; Muller et al., 2021; Zhuo et al., 2021). Similarly, the frontoparietal control network—indexed primarily by the superior and middle frontal gyri, and the salience network, captured by the anterior insula and rostral cingulate cortex, demonstrated substantial abnormal loads. These effects were especially pronounced in longitudinal designs and in studies stratifying patients by symptom severity or clinical phenotype (Niu et al., 2020; Burrowes et al., 2020; Dean and Sterr, 2015; Muller et al., 2021).

Analysis of the normalised abnormality-per-region index (A_k_) indicated that the DMN and limbic/memory networks were characterised by both a high number of affected regions and a high frequency of abnormalities per region, suggesting consistent network-level vulnerability. In contrast, the frontoparietal and salience networks involved fewer distinct regions but were nevertheless implicated with notable consistency across studies. Sensorimotor, interhemispheric (callosal), and cerebellar/brainstem networks contributed at a secondary yet non-negligible level, particularly in studies examining more severe injury profiles or chronic mTBI cohorts (Zhou et al., 2013; Muller et al., 2021; Kim et al., 2021).

### Co-alteration structure

Analysis of the co-alteration matrix and the corresponding graph revealed a structured, non-random pattern of regional co-involvement. When co-occurrence was restricted to regions reported as abnormal in at least two independent studies, the resulting graph was characterised by a dense core module linking the precuneus, posterior and mid cingulate regions, medial temporal structures, the thalamus, and selected frontal areas. This constellation forms a tightly interconnected DMN–limbic–relay circuit, whose constituent regions tend to be affected together across different cohorts, post-injury time points, and analytic approaches.

**Figure 3:**
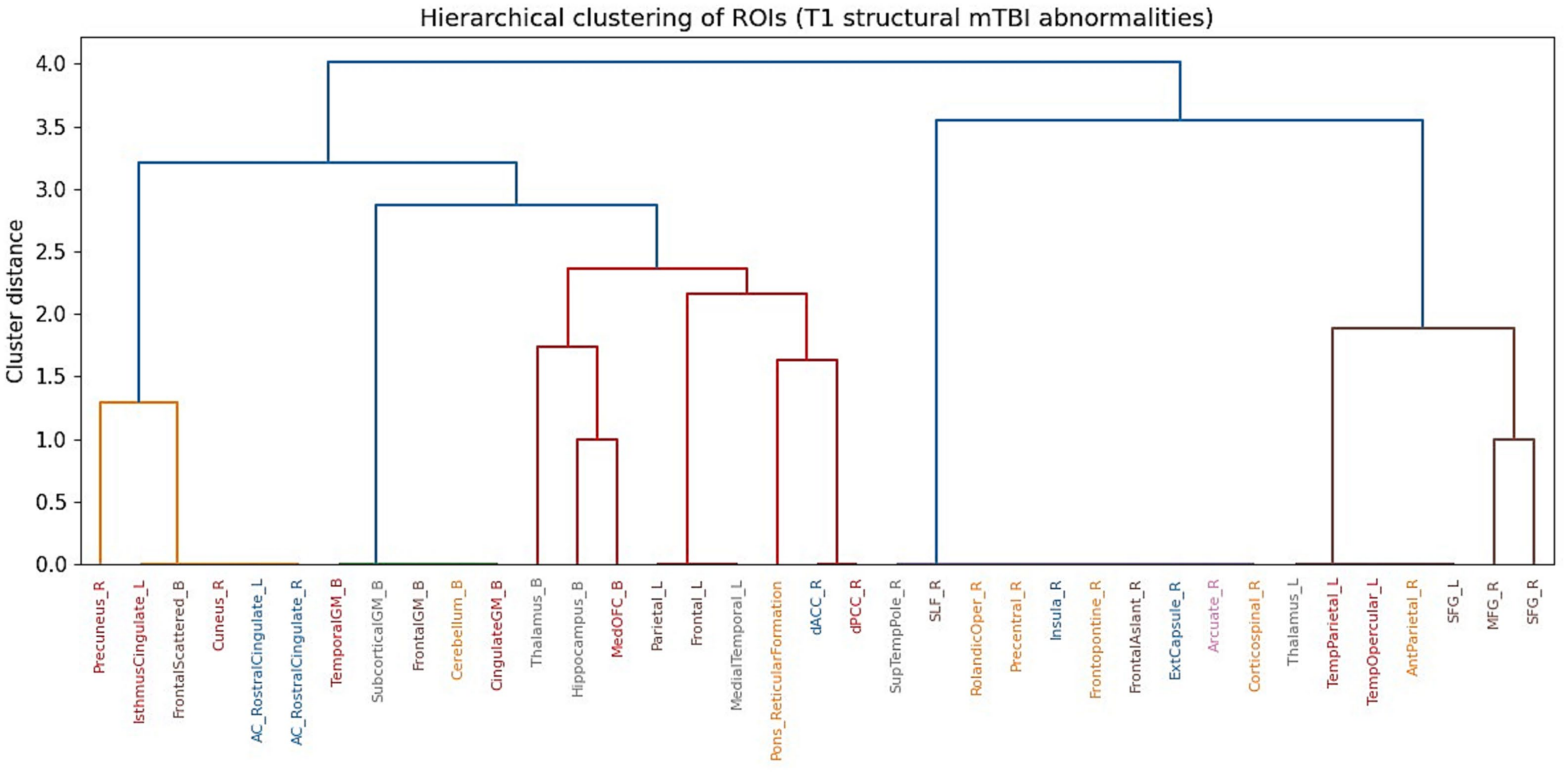
Hierarchical clustering of T1-weighted structural abnormalities in mTBI. Dendrogram showing the hierarchical similarity of structural abnormality profiles across ROIs using Ward’s clustering method. Labels are color-coded by canonical network. The earliest branching cluster contains precuneus, posterior and mid cingulate, hippocampus, and thalamus, reflecting a DMN–limbic structural core. Subsequent clusters correspond to frontoparietal/salience networks, followed by sensorimotor and brainstem/cerebellar regions. This alignment between data-driven clustering and established neurocognitive networks reinforces the network-organised nature of structural injury in mTBI and validates the Structural Network Fingerprint (SNF) framework.

A second, partially overlapping module comprised the anterior insula, superior and middle frontal gyri, and temporal opercular regions. This cluster aligns with a frontoparietal–salience system implicated in attentional control, interoceptive processing, and cognitive–emotional integration. The observed co-alteration structure suggests that mTBI-related structural abnormalities do not arise solely through local anatomical proximity, but rather follow patterns of functional and network-level integration. Hierarchical clustering of ROIs based on their abnormality profiles revealed modular organization broadly consistent with canonical network assignments (Figure 3).

### PCA and hierarchical clustering

Principal component analysis of the region-by-study matrix demonstrated that the first principal component accounted for a substantial proportion of the variance and primarily differentiated DMN–limbic regions from more lateral sensory and motor areas. Regions including the precuneus, posterior cingulate cortex, hippocampus, and thalamus exhibited strong and concordant loadings on the first component, consistent with their shared pattern of vulnerability across studies. The second principal component distinguished prefrontal cortical regions and association white matter tracts from subcortical and brainstem structures, suggesting a hierarchical axis of structural involvement extending from cortical control systems to deeper relay regions.

Hierarchical clustering of regions based on their abnormality profiles across studies produced clusters that broadly mirrored established large-scale network assignments. DMN and limbic regions clustered early, followed by groups dominated by frontoparietal and salience regions, while brainstem and cerebellar regions formed later, more distinct branches. The close correspondence between these data-driven clusters and canonical network labels provides convergent support for the validity of the structural network fingerprint framework.

### Sensitivity and robustness analyses

To evaluate the stability of the Structural Network Fingerprint (SNF), additional analyses were performed using alternative co-alteration thresholds (*τ* = 1 and *τ* = 3). Lowering the threshold to *τ* = 1 increased graph density but preserved the central DMN–limbic–thalamic module, indicating that the core structural constellation was not dependent on threshold stringency. Increasing the threshold to *τ* ≥ 3 resulted in a sparser network; however, the same medial and limbic regions remained interconnected, supporting robustness of the principal module.

We additionally explored alternative network grouping strategies, including separation of frontoparietal and salience systems rather than partial aggregation. Across configurations, the Default Mode Network and limbic/memory network consistently demonstrated the highest abnormal load and abnormality-per-region indices. These findings indicate that the primary fingerprint structure is stable across reasonable analytic variations and is not an artifact of specific thresholding or grouping choices.

## Discussion

In this study, we introduce a mathematically explicit Structural Network Fingerprint (SNF) for mild traumatic brain injury derived exclusively from T1-weighted structural MRI. By systematically extracting region-level abnormalities from ten independent studies, harmonising them within a shared anatomical and network framework, and applying matrix-based and graph-theoretical analyses, we demonstrate that mTBI is associated with a reproducible pattern of structural vulnerability organised at the level of large-scale brain networks.

A central finding of this synthesis is the prominence of a DMN–limbic–thalamic core. Regions including the precuneus, posterior and mid cingulate cortex, hippocampus, and thalamus emerged repeatedly across studies, consistent with extensive prior work implicating these structures in self-referential processing, memory, arousal, and fatigue (Buckner et al., 2008; Mayer et al., 2017; Zhuo et al., 2021; Iraji et al., 2015). Among these, the thalamus appears to play a particularly critical role. Structural and functional alterations of thalamic circuits have been linked to long-term outcome following mTBI, reflecting its position as a key relay and integrative hub (Mavroudis et al., 2022; Xiong et al., 2016). Our findings extend this literature by showing that thalamic volume loss reliably co-occurs with abnormalities in DMN and limbic regions across diverse cohorts and analytic approaches, reinforcing its centrality in the network-level pathophysiology of mTBI.

### Conceptual distinction from coordinate-based meta-analysis

The Structural Network Fingerprint (SNF) framework should be understood as complementary to, rather than a substitute for, coordinate-based meta-analytic approaches such as ALE. Whereas ALE quantifies spatial convergence of reported coordinates across studies, SNF preserves study-specific regional profiles and explicitly encodes the direction of abnormality. This distinction allows SNF to characterize not only where abnormalities converge, but also how they co-occur across distributed systems and whether they tend toward structural increase or decrease.

Furthermore, by assigning regions to canonical large-scale networks, SNF shifts the analytic focus from focal peak overlap to network-level vulnerability. The resulting representation supports graph-theoretical modeling, dimensionality reduction, and clustering analyses that reveal modular organization and hierarchical structure within the aggregated dataset. In this sense, SNF emphasizes systems architecture and reproducible co-alteration patterns rather than coordinate density alone. Importantly, SNF does not provide pooled effect size estimates nor formal statistical inference across coordinates. Instead, it offers a mathematically explicit synthesis framework that preserves interpretability at the level of named anatomical regions and functional networks, thereby facilitating integration with computational modeling and future patient-level deviation analyses.

The involvement of frontoparietal and salience networks further aligns with the clinical phenotype of post-concussive syndrome, in which impairments of attention, executive function, and emotional regulation are prominent (Mayer et al., 2017; Silverberg and Iverson, 2013; van der Horn et al., 2020). Structural abnormalities affecting the superior and middle frontal gyri, anterior insula, and temporal–opercular regions likely contribute to these deficits and mirror alterations in functional activation and connectivity reported in other neuroimaging modalities (Mayer et al., 2012; King et al., 2019; Hillary et al., 2014). Notably, the co-alteration patterns identified here suggest that disruption of these control networks does not occur in isolation but is closely coupled with damage to midline DMN–limbic hubs, pointing to a coordinated, network-level mode of injury.

The SNF framework differs from conventional meta-analytic approaches in several important respects. First, rather than treating activation likelihood estimation (ALE) clusters as independent observations, we returned to the primary T1-weighted studies and extracted their reported regional findings directly, thereby preserving study-level heterogeneity and avoiding double-counting of evidence. Second, by operating at the level of anatomically defined regions and mapping them onto canonical functional networks, the framework establishes an explicit link between structural injury and functional systems. Third, the representation of abnormalities in a region-by-study matrix, coupled with straightforward yet rigorous network metrics, co-alteration graphs, and dimensionality-reduction techniques, yields a computationally tractable approach that can be readily implemented in software environments.

From a clinical and translational perspective, the present findings should be interpreted with appropriate caution. The Structural Network Fingerprint (SNF) described here is derived exclusively from aggregated, study-level data and does not constitute an individual-level diagnostic model. Accordingly, the current framework should not be interpreted as directly applicable to patient-level diagnosis, prognostication, or medicolegal adjudication. Rather, the SNF provides a structured systems-level synthesis of convergent T1-weighted findings across heterogeneous cohorts. Its principal contribution lies in identifying reproducible patterns of network-level structural vulnerability that may inform future hypothesis-driven investigations.

In principle, extension of this framework to individual-level modeling would require standardized parcellation pipelines, normative reference datasets, prospective validation cohorts, and formal predictive modeling approaches. Such developments remain beyond the scope of the present study and represent important directions for future research. The present results should therefore be understood as establishing a computational and conceptual scaffold upon which future patient-level network deviation metrics may be developed and rigorously validated.

An important consideration in interpreting the present findings concerns the heterogeneity of reported structural directionality. While the dominant pattern across studies consisted of regional volume reductions, several investigations reported localized grey-matter increases, particularly within cingulate regions. The biological meaning of such increases remains uncertain.

T1-weighted volumetric expansion does not necessarily imply enhanced neuronal integrity or functional improvement. Potential mechanisms include transient neuroinflammatory processes, gliosis, vascular or interstitial fluid changes, compensatory dendritic remodeling, or methodological variability in segmentation and normalization procedures. Moreover, the direction of structural change may depend critically on post-injury timing, symptom profile, or cumulative injury burden.

Accordingly, caution is warranted in equating volume loss with irreversible neurodegeneration or volume increase with adaptive compensation. The SNF framework encodes directionality descriptively, without imposing mechanistic interpretation. Its purpose is to characterize reproducible patterns of convergence across studies, while acknowledging that the underlying biological substrates of T1-derived morphometric change are likely multifactorial and temporally dynamic. Future longitudinal and multimodal investigations integrating structural MRI with inflammatory markers, diffusion imaging, and functional connectivity measures will be necessary to clarify the biological drivers of directional heterogeneity observed in mTBI.

### Methodological limitations and sources of heterogeneity

Several limitations of the present synthesis warrant explicit consideration. First, the SNF framework is derived from a modest number of primary studies (*n* = 10), reflecting the relatively limited availability of T1-weighted morphometric investigations meeting strict inclusion criteria. Although convergence across independent cohorts strengthens confidence in the identified network-level pattern, the fingerprint should not be interpreted as exhaustive of all structural alterations in mTBI.

Second, substantial heterogeneity exists across the included studies with respect to post-injury timepoints, clinical phenotype, symptom severity, and study design. Some cohorts were examined in the acute or subacute phase, whereas others focused on chronic or longitudinal follow-up populations. Given that structural alterations may evolve dynamically over time, the present SNF likely aggregates phase-specific processes into a single composite representation. Future work should examine whether temporally stratified fingerprints (e.g., acute versus chronic mTBI) exhibit distinct topological characteristics.

Third, cohort composition varied across studies, including differences in age distribution, injury mechanism (e.g., sport-related concussion versus civilian trauma), and presence of persistent post-concussive symptoms or post-traumatic headache. These factors may influence both the spatial distribution and directionality of structural abnormalities.

Fourth, as with all syntheses based on published findings, the present framework is potentially subject to reporting bias. Regions not reaching statistical significance in individual studies are typically not reported, and null findings are less likely to be published. Consequently, the region-by-study matrix reflects reported abnormalities rather than a fully sampled anatomical landscape.

Finally, the SNF does not incorporate quantitative effect sizes, nor does it weight findings by sample size or study quality. While this choice preserves methodological comparability and avoids invalid cross-study pooling, it limits inferential precision. The framework therefore emphasizes reproducible topological convergence rather than magnitude estimation.

Taken together, these limitations underscore that the SNF represents a structured aggregation of heterogeneous study-level evidence. Its strength lies in identifying reproducible network organization across diverse datasets, but its conclusions should be interpreted within the context of methodological variability and temporal heterogeneity inherent to the mTBI literature.

## Conclusion

By integrating T1-weighted structural MRI findings from ten independent mTBI studies into a unified region-by-study representation and interrogating this matrix using network-informed metrics, co-alteration modeling, principal component analysis, and hierarchical clustering, we identified a reproducible Structural Network Fingerprint of mild traumatic brain injury. This fingerprint is characterized by a convergent DMN–limbic–thalamic core, with additional involvement of frontoparietal and salience systems, and secondary contributions from sensorimotor and brainstem structures. The observed organization suggests that structural abnormalities in mTBI preferentially affect highly interconnected network hubs rather than isolated anatomical regions.

Importantly, the present framework represents a structured synthesis of study-level evidence rather than a validated clinical instrument. Its primary contribution lies in formalizing network-level convergence in a mathematically explicit manner and demonstrating the reproducible topological organization of T1-based abnormalities in mTBI.

Future work should evaluate phase-specific fingerprints (acute versus chronic injury), incorporate multimodal imaging data, and test whether individual-level deviation metrics derived from this framework exhibit diagnostic or prognostic utility in prospective cohorts. Until such validation is achieved, the SNF should be regarded as a conceptual and computational research framework rather than a clinical decision-support tool.

## Data Availability

The original contributions presented in the study are included in the article/supplementary material, further inquiries can be directed to the corresponding author/s.
